# How do researchers perceive problems in research collaboration? Results from a large-scale study of German scientists

**DOI:** 10.3389/frma.2023.1106482

**Published:** 2023-02-23

**Authors:** Carina Weinmann, Malte Hückstädt, Florian Meißner, Gerhard Vowe

**Affiliations:** ^1^Department of Social Sciences, Heinrich Heine University of Düsseldorf, Berlin, Germany; ^2^Research Area Governance in Higher Education and Science, German Centre for Higher Education Research and Science Studies (DZHW), Berlin, Germany; ^3^Faculty of Culture, Media, Psychology, Macromedia University of Applied Sciences, Cologne, Germany; ^4^Department of Ethical, Legal and Social Implications, Center for Advanced Internet Studies (CAIS), Bochum, Germany

**Keywords:** research collaboration, research teams, collaboration problems, representative survey, differences

## Abstract

In recent years, collaboration has become the norm in scientific knowledge production. Like other forms of collaboration, research collaborations (RCs) face specific problems that can jeopardize success. Against this background, the present study sought to gain a deeper understanding of the relevance of different collaboration problems and the interconnections among these problems. Building on previous insights into the most current problems, we addressed four major issues: (1) researchers' perceived relative relevance of collaboration problems in their projects (in terms of their occurrence), (2) differences in these perceptions based on the type of RC (e.g., number of subprojects and collaboration mode) and (3) on the characteristics of researchers, and (4) the co-occurrence of collaboration problems. Based on a representative survey of leading participants of RCs funded by the German Research Foundation (*n* = 5,326), we found that researchers experienced collaboration problems (e.g., fairness and communication problem) only to a small degree, and there were almost no differences regarding their perceived relevance. Furthermore, there were almost no significant differences concerning the perceived relevance of these problems depending on the type of RC or the individual researchers. However, the findings did reveal specific patterns of co-occurrence (e.g., relationship and difference problem). The results suggest that previous research may have overstated the relevance of collaboration problems in RCs. Instead, it seems that at least in Germany, collaborative research works better than one might assume.

## 1. Introduction

To an ever growing degree, scientific knowledge is the product of collaborative research (Baker et al., 2017). However, when substantial problems arise in the interaction between members of a research collaboration (RC), the success of the collaboration is at risk (Bozeman et al., 2016; Sacco, 2020). To foster the understanding of which problems are particularly important for RCs, this paper builds on previous work, which compiled a theoretically and empirically informed catalog of seven typical problems that research teams experience (Meißner et al., 2022). These (internal) problems refer to difference, commitment, certainty, communication, fairness, management, and relationship aspects. However, there is still little empirical evidence concerning the relative importance—and the interconnectedness—of these problems, let alone with respect to potential differences between different types of RCs.

To enable successful RCs, it is crucial to gain a full picture of the social dynamics within RCs and the challenges they face. This is illustrated by the strong dependence of RCs on reciprocal and trustful interactions between their members: “Collaborative research teams are defined as largely voluntary, substantially autonomous, self-governed social entities or systems based on mutual interest of multiple individuals” (Kosmützky, 2018). Hence, when a collaboration becomes problematic, there is no external force that can solve any particular problem. Instead, the team members have to solve problems on their own—or prevent them from occurring in the first place. If, for instance, one or more members lack the commitment that is needed to achieve the RC's goals, the other members might be able to compensate for this problem to a certain degree. But if there is not even a minimum level of reciprocity between all members, the overall performance of the RC will deteriorate. In such cases, the social relationships within the RC can easily become toxic, further jeopardizing the achievement of the RC's goals. Finally, it is also important to look at the specifics of different types of RCs, because the constellations of problems may differ. For example, larger collaborations with many subprojects might face more problems with respect to communication and differences. The same might be the case for university-industry collaborations compared to academic-only RCs.

Against this background, we formulated the following leading research question: *How do researchers perceive the relevance of problems in research collaboration, and how are these problems interconnected?*

We aimed to answer this question by presenting data from a representative survey of leading researchers (i.e., spokespersons and principal investigators) who participated in RCs funded by the German Research Foundation. The latter is the prime research funding body in Germany. The fact that the research team was granted access to the database of the German Research Foundation was a unique research opportunity, allowing for a survey among thousands of scientists conducting collaborative research under the different schemes offered.

The paper is structured as follows: First, we describe the current state of research, which yields a fragmented picture of the hierarchies of relevance of problems in RCs. Second, based on previous research, we provide a detailed outline of the seven typical problems in research collaboration. Third, we derive our research questions, aiming to achieve an understanding of the relative importance and interconnectedness of problems in RCs. Fourth, we describe the sample and methods used. Fifth, we present our results, which demonstrate that collaboration problems are generally experienced only to a small degree. Finally, we discuss the results, concluding that problems in RC are less prevalent than may have been expected based on previous study findings.

Our understanding of research collaborations (RCs) is that of inter- or intraorganizational teams of researchers who work together in a limited time frame and in order to achieve a common research objective. In most cases, their work is funded by external resources (e.g., Katz and Martin, 1997; Bozeman et al., 2013; Kosmützky, 2018). Previous research suggests that “most research collaborations succeed and most research teams are ‘happy ones”' (Bozeman and Youtie, 2017). Nevertheless, a large number of studies have revealed a range of problems that may occur in collaborative research. Among the most frequently mentioned problems are various types of differences, such as different institutional logics (e.g., Cummings and Kiesler, 2007; Bjerregaard, 2010; Garcia et al., 2019), gender and cultural differences (e.g., Barnes et al., 2002; Bozeman and Gaughan, 2011; Abramo et al., 2013), status and role conflicts (e.g., Hackett, 2005; Bendersky and Hays, 2012; Youtie and Bozeman, 2014), and ineffective or insufficient communication (e.g., Barnes et al., 2002; Cohen et al., 2011; Wöhlert, 2020). However, the majority of studies on this issue focused either on specific problems or specific types of RC such as university-industry collaborations (e.g., Barnes et al., 2002) or research teams from specific disciplines (e.g., Bozeman et al., 2016). While a synopsis of these studies might thus lead to a broad list of potential collaboration problems, scientific knowledge about problems of RC in general, about the relevance of specific problems and in specific contexts, and about the interconnections between them is rather scarce. Furthermore, prior research aims to understand what might be collaboration problems and how to solve them rather than providing knowledge about their actual occurrence.

Taking these shortcomings into account, a recent study aimed to identify the most important problems in collaborative research in general (Meißner et al., 2022). The authors adopted a micro-theoretical view, implying an emphasis on the perspective of the researchers involved and their ability to solve collaboration problems on their own. They conducted in-depth interviews with researchers from a range of RCs, including different disciplines and types of collaboration (e.g., with respect to their size, collaboration mode, and funding agency). From these interviews as well as from an extensive literature review, seven main collaboration problems emerged, which the authors labeled and, based on the frequency of mentions and the ascribed importance, weighted with respect to their perceived relevance across all interviewees (Table 1).

**Table 1:** Collaboration problems and their perceived relevance (Meißner et al., 2022).

**Label**	**Description**
**High relevance**	
Difference problem	Too large differences between members of a RC (e.g., differences with respect to motivations and objectives)
Commitment problem	A substantial proportion of research team members are rather focused on their own research domain at the cost of the collective interests of the RC (e.g., exploitation of a RC in financial terms)
Certainty problem	Unforeseeable uncertainties and risks are a burden for the collaboration (e.g., dropout of a collaboration partner)
**Medium relevance**	
Communication problem	Insufficient and/or one-sided interaction and communication between membership and leadership (e.g., leadership does not include members in discussions)
Fairness problem	Unfair distribution of individual inputs and outcomes (e.g., co-authorship, distribution of personal, financial, and technical resources)
Management problem	Incompetence in a RC's leadership (e.g., participants in leadership positions take advantage of their status rather than focusing on the interests of the RC as a whole)
**Low relevance**	
Relationship problem	Problematic personal relationships that strain the work process (e.g., social ties are perceived as too weak or dysfunctional)

The identification of these problems enables a detailed and differentiated understanding of the most common collaboration problems. Additionally, the study offers a generalizable list of potential problems in collaborative research and provides reasonable evidence for their conciseness and comprehensiveness (Meißner et al., 2022). This list therefore serves as the basis for the aim of the present paper. However, from a quantitative perspective, the informative value is still limited. First of all, given the qualitative nature of the study and the reliance on a relatively small sample, the robustness of the problem hierarchy is limited. We therefore aimed to assess whether the perceived relevance of collaboration problems found can be validated in terms of their actual occurrence. For example, are differences between members of a RC actually perceived as more problematic than an incompetent leadership? To the best of our knowledge, there are no quantitative findings supporting this argument. Therefore, we posed the following research question:

*RQ1: How does the perceived relevance of the seven collaboration problems differ?*

Research collaborations considerably differ from one another, for example, with respect to the involved disciplines, team sizes, and team constellations. Accordingly, the actual relevance of the seven problems might vary depending on the type of collaboration. Specifically, the findings by Meißner et al. (2022) indicated that the perceived relevance of problems is different in academic-only RCs as compared to university-industry collaborations. For instance, it is feasible that the difference problem may be less relevant in RCs containing only researchers with an academic background. As intercultural teams require high communicative efforts to negotiate and align different expectations (Gläser et al., 2015), the communication problem might be of high relevance in such teams. Other problems, such as the management problem, might be more likely to occur in larger research teams than in smaller ones (Vonortas and Spivack, 2006; Cummings et al., 2013). And problems such as the relationship problem might be more likely to occur at the beginning of the collaboration than later on. Again, previous research offers little insight into this issue. Therefore, we posed the following research question:

*RQ2: How does the perceived relevance of collaboration problems differ depending on the type of RCs with respect to the number of disciplines involved, duration, number of subprojects, collaboration mode, and disciplinary composition?*

Individual backgrounds might also give rise to substantial differences in the perceived relevance of collaboration problems. Different employment positions most certainly lead to different perceptions of when and to what extent collaborative research becomes problematic. Due to the responsibilities of their role, for instance, spokespersons of RCs might attribute more relevance to the certainty problem compared to other team members such as principal investigators. Differences can also be expected based on the disciplinary affiliation of researchers. For example, due their disciplinary culture, researchers from the humanities might experience uncertainty as less problematic then researchers from other fields such as engineering. As with the questions raised above, research findings to date only allow for speculations on this matter. Our third question was therefore:

*RQ3: How does the perceived relevance of collaboration problems differ depending on individual characteristics of researchers with respect to their disciplinary affiliation and employment position?*

In reality, collaboration problems may only rarely occur alone; rather, specific problems may co-occur or mutually influence each other (Meißner et al., 2022). For example, it is highly plausible that the management problem triggers other problems due to the central role of competent leadership for successful collaboration (Bozeman et al., 2016). As another example, the difference problem might be accompanied by a commitment problem, because different backgrounds such as university vs. industry result in divergent expectations regarding the necessary efforts (Barnes et al., 2002). Similar to the question of the relevance of the respective problems, empirical knowledge about the co-occurrence of problems is currently lacking. However, this knowledge is of central relevance, not only with respect to the problems themselves but also because knowledge about which problems co-occur may also inform the specific problem-solving strategies. Thus, our fourth research question is:

*RQ4: Which perceived collaboration problems frequently co-occur?*

## 2. Materials and methods

### 2.1. Participants and procedure

Cross-sectional data were used to test the hypothesis and answer the research questions. The data were obtained through a web survey conducted in 2020 as part of the collaborative research project Determinants and effects of cooperation in homogeneous and heterogeneous research clusters (DEKiF). The main focus of the web survey was on collaboration problems in RCs and the solutions to such problems. The survey was sent to *N* = 15,595 spokespersons and principal investigators (PIs) from RCs of Coordinated Programmes^1^ (German Research Foundation, 2017), Excellence Initiatives (German Research Foundation, 2019), and the Excellence Strategy (German Research Foundation, 2020) that were currently ongoing or had been completed after 2015 (Supplementary Figure A1 in Supplementary material). During the 7-week field phase, one invitation and four reminders were sent out to the target persons. A total of *n* = 3,875 participants completed the survey in full, while *n* = 1,451 participants completed the survey partially. The overall total of *n* = 5,326 participants amounted to a response rate (RR2) of 34.15%. No contact could be initiated with 4.27% of the target persons due to outdated email addresses. The sample consisted of 26% females and 74% males. As only 0.001% of the respondents belonged to diverse gender, these were excluded from the analyses due to the small group size. The mean age of the respondents was $\bar{x}$ = 52.67 years with a standard deviation of *SD* = 9.52.

According to the German Research Foundation, the submission of a statement by an ethics committee is required if patients or persons with special protection needs are involved. The submission of an ethics approval is also required if a study involves physical risks to the participants, potential participants are not to be informed of a study, participation in a study involves deception, or if a study exposes participants (interviewees, those providing information, project staff, researchers, and research subjects) to exceptional risks (German Research Foundation, 2022). Because none of these cases applied to our study we did not seek approval from an ethics committee. The participants were asked to give informed consent as part of the questionnaire (i.e., written form). The text for the informed consent had been reviewed by the responsible data protection officer.

The RC of the funding lines, coordinated programmes (German Research Foundation, 2023b) and clusters of excellence (German Research Foundation, 2023c) comprise a wide range of differently constituted clusters: our sample includes inter- and intraorganisational, mono-, multi-, cross- inter- and transdisciplinary cooperating research clusters,^2^ of different sizes in terms of personnel, heterogeneous in terms of disciplines, spatially distributed and of different durations (Supplementary Figures A2–A6 in Supplementary material). Beyond the different, overarching funding priorities of the targeted funding lines (such as, for example, the development of a scientific profile at the location of the applicant Higher Education Institutions or the establishment of internationally visible and competitive research institutions), what all RCs have in common is that they fund the work on ambitious, complex and long-term research projects. In this context, the German Research Foundation places central importance on close (often interdisciplinary) cooperative relationships between the PIs of an RC, which are directed toward the production of research results that clearly exceed the possible achievements of individual researchers or subprojects. In this respect, the PIs of an RC must cooperate closely on an ongoing basis in order to achieve their common research goals, fulfill their overarching function and thus ensure the continued existence of their RC (Defila et al., 2006).

The population we are addressing is interesting not only because it contains PIs and spokepersons from different disciplines who work closely together under different conditions in RCs of different sizes and with varying disciplinary heterogeneity, thus decisively shaping the realities of long-term research collaborations. The choice of the population was also methodologically motivated: Before the survey was conducted, a complete list of contact addresses of all target persons in the population was generated via the GEPRIS database (German Research Foundation, 2021), which made it possible to aim for a full survey and to obtain a sample that was robust in terms of inferential statistics (Toepoel, 2016). Due to the availability of a list of all target persons in the population, the difference between the inferential population, the target population and the sampling frame was eliminated (Weisberg, 2009). Biases in the representation of the sample could therefore only occur through unrealizable contacts (see above) and through unit non-response. A unit non-response analysis (Weisberg, 2009) of the sample showed that the non-response error is low with regard to the (1) professional affiliation and the (2) gender of the PIs and directors as well as with regard to their (3) affiliation to current and terminated collaborations (4) of the various funding lines: The relative frequencies of the characteristics of the variables mentioned deviate on average 1.9% in the sample from those in the population (see Supplementary Figures A7, A8 in Supplementary material). In this respect, it is assumed that the following analyses are based on a statistically reliable sample.

Finally, it should be noted that the research project Determinants and effects of cooperation in homogeneous and heterogeneous research clusters (DEKiF) was neither funded by the DFG nor supported by it in any way except by granting access to its collaborative research project database. The target persons were informed that the survey and metadata obtained would be used exclusively by the DEKiF project for scientific purposes, that individual persons and RC could not be identified from published results and that the results would not be exclusively communicated back to the DFG in any way.

### 2.2. Measures

To measure the perceived relevance of collaboration problems, we constructed two items for each of the seven collaboration problems. Respondents rated their agreement with the fourteen items displayed in Table 2 on a 5-point Likert scale from 1 = “Not at all” to 5 = “Completely.” The items were conceived based on the research literature on collaboration problems and on analyses of *N* = 18 expert interviews with spokespersons and PIs (Meißner et al., 2022). All numerical values of the fourteen Likert-scaled items were inverted before analysis, such that an increase in value of an item can be interpreted as an increase in the relevance of the respective problem dimension. To reduce the complexity of the item battery, we calculated a cumulative index of the two items of each of the seven problem dimensions (Spearman-Brown coefficient > 0.6; Table 2).

**Table 2:** Items measuring the problems.

**Items**	**Spearman-Brown correlation**	**Problem**
“*The collaboration^*a*^ members reliably engage in the RC*.”		
“*The collaboration members collaborate toward the achievement of the common research objectives, also beyond the boundaries of subprojects*.”	*r* = 0.78	Commitment problem
“*The costs and benefits of RC work are shared fairly between the members*.	*r* = 0.80	Fairness problem
*The collaboration members' contributions to achieving the common research objectives are appropriately recognized at the collaboration level.”*		
“*The communication at the collaboration level^*b*^ is comprehensive.”*	*r* = 0.84	Communication problem
“*The collaboration members participate actively in communication within the RC.”*		
“*There is sufficient agreement among collaboration members on the common objectives at the collaboration level.”*	*r* = 0.78	Difference problem
“*The collaboration members are able to overcome discipline-related differences.”*		
“*The collaboration at the collaboration level is characterized by mutual trust.”*	*r* = 0.80	Relationship problem
“*The different working styles of the collaboration members are compatible.”*		
“*The spokesperson is primarily oriented toward the interests of the RC as a whole.”*	*r* = 0.64	Management problem
“*The spokesperson grants the PIs a sufficient degree of autonomy.”*		
“*The RC members do everything in their power to reliably deliver their contributions to achieving the common RC objectives.”*	*r* = 0.71	Certainty problem
“*The RC is adequately prepared for the fact that delays or unforeseen situations may occur in the research process.”*		

^a^For ease of understanding, we depart here from the original survey terms and call “collaborations” “RC”.

^b^In the context of the survey, the collaboration level was understood as the level on which the spokesperson and the PIs work together across subprojects to achieve the common collaboration goals.

To analyze potential group differences in the intensity of collaboration problems, we specified eighteen subsamples based on the following seven group variables^3^: (1) the *number of disciplines involved*,^4^ (2) the *duration of the RCs*, (3) the *number of their subprojects*, (4) their *collaboration mode*, (5) their *disciplinary composition*, (6) the individual *disciplinary affiliation*, and (7) the *employment position* of the respondents.^5^ The values of the numerical variables (1), (2), and (3) were each assigned to one of three categories (see Figures 2, 3). The following analyses are based on the cumulative indices (from 2 to 10).

### 2.3. Analytical procedures

To answer RQ1–RQ3, we report the arithmetic mean values of the cumulative indices of the seven problem dimensions. To answer RQ2 and RQ3, a total of seven variables were used to reveal and test possible group differences in the perception of collaboration problems. These are (1) the *number of disciplines involved*, (2) the *duration of the RCs*, (3) the *number of their subprojects*, (4) *collaboration mode*, (5) their *disciplinary composition*, (6) the individual *disciplinary affiliation*, and (7) the *employment position* of the respondents.

#### 2.3.1. Permutational MANOVA

The possible differences in mean values between the groups of the total sample and the cumulative indices of the seven problems could not be tested for statistical stability using a traditional multivariate analysis of variance (MANOVA) because classical MANOVA assumes that the dependent variables are normally distributed (Anderson, 2001). As this was not the case for the cumulative indices of the seven problems, we used permutational multivariate analysis of variance (Anderson, 2001) (PERMANOVA) to answer RQ2 and RQ3. In contrast to traditional MANOVA, the decomposition and calculation of the variance of the subgroupings in PERMANOVA is based on a semimetric or metric dissimilarity matrix. Similar to the classical MANOVA, the *pseudo F-ratio* is the central test statistic in the PERMANOVA framework.

Accordingly, the total sum of squared dissimilarities of objects belonging to different groups is compared with the squared dissimilarities of objects belonging to the same group. The more the groups differ in terms of the total sum of squared dissimilarities, the higher the *pseudo-F ratio*. To calculate the significance of the pseudo F-ratio, PERMANOVA uses a permutation procedure: “The *p*-value is calculated from the proportion of permuted pseudo F-statistics which are greater than or equal to the observed F-statistic. (...) If more than 5% of the permuted F-statistics has values greater than that of the observed F statistic, the p-value is greater than 0.05” (Xia et al., 2018). As a result of the non-significant permutation test of the PERMANOVA, it can be concluded that the differences between the specified groups were not statistically significant.

#### 2.3.2. Multidimensional scaling

To answer RQ4, we specified an ordinal multidimensional scaling (MDS) model (König, 2017; Borg et al., 2018) based on Pearson correlations between the seven collaboration problems. The MDS allowed us to construct a “perceptual space” (Kruskal and Wish, 2009), which visualized the overall structure of the correlations between the seven collaboration problems, thus enabling us to identify problem patterns.

The goal of MDS is to optimally reflect the original proximity information of the study objects in a low-dimensional space. In this process, the study objects are iteratively configured to each other in a geometric space until the fit between the global solution of the MDS and the proximity information of the input material can no longer be further optimized. The global fit of the geometric solution of the MDS is quantified using the sum of squared fitting errors: the stress value. The smaller the stress value of a geometric solution of an MDS model, the better its fit (Borg et al., 2018). The interpretation of an MDS solution is intuitive:^6^ The higher the correlation of two objects, the smaller their distance in the geometric space of the MDS model. Consequently, if two objects are located far apart, they are in essence uncorrelated. Furthermore, the points that are located in the center of the point cloud are positively correlated with all other objects. Therefore, items that are located closer to the periphery of the MDS configuration are positively correlated with items in their neighborhood, but not with items opposite to them (Borg et al., 2018). Finally, it is important to note that the dimensions of the resulting MDS configuration do not have substantive meaning *per se*. Accordingly, an absolute interpretation of the positions of the objects in the geometric space of the MDS model is not reasonably possible.

## 3. Results

### 3.1. Overall relevance of the collaboration problems (RQ1)

**Figure 1:**
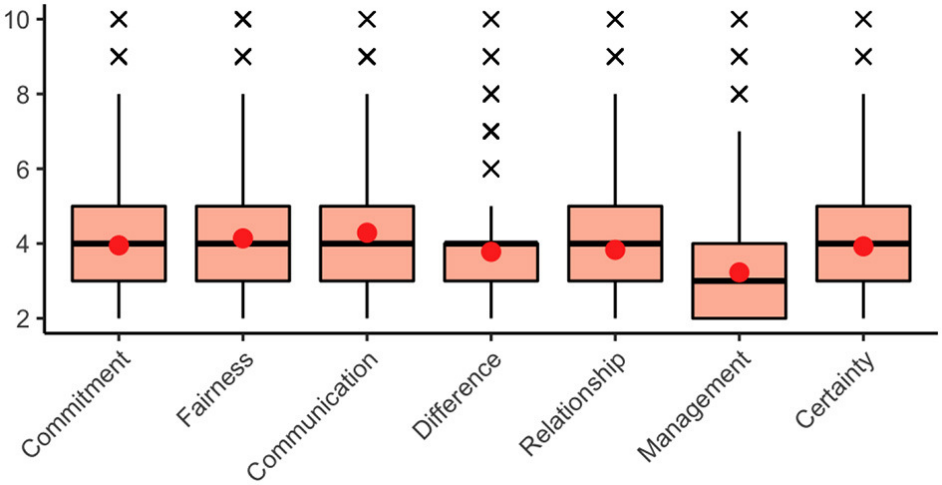
Perceived relevance of the seven collaboration problems. The arithmetic mean value of the cumulative indices is shown as a red dot, the median as a thick horizontal line. Outlier values are represented by black crosses beyond the boxes. The graph is based on the survey data of *n* = 3,050 PIs and spokespersons after listwise deletion.

The global relevance ratings of the collaboration problems (RQ1) by the PIs and spokespersons revealed that RCs generally only experience collaboration problems at the collaboration level to a small degree: The mean values for commitment, fairness, communication, difference, relationship, and certainty problems lay at around *M* = 4. These six problems were thus evenly scaled to have little relevance for both the PIs and spokespersons.^7^ Of even less relevance to the PIs and spokespersons were management problems, with a mean value of *M* = 3.23 (see Figure 1).

### 3.2. Differences depending on the type of RCs (RQ2)

**Figure 2:**
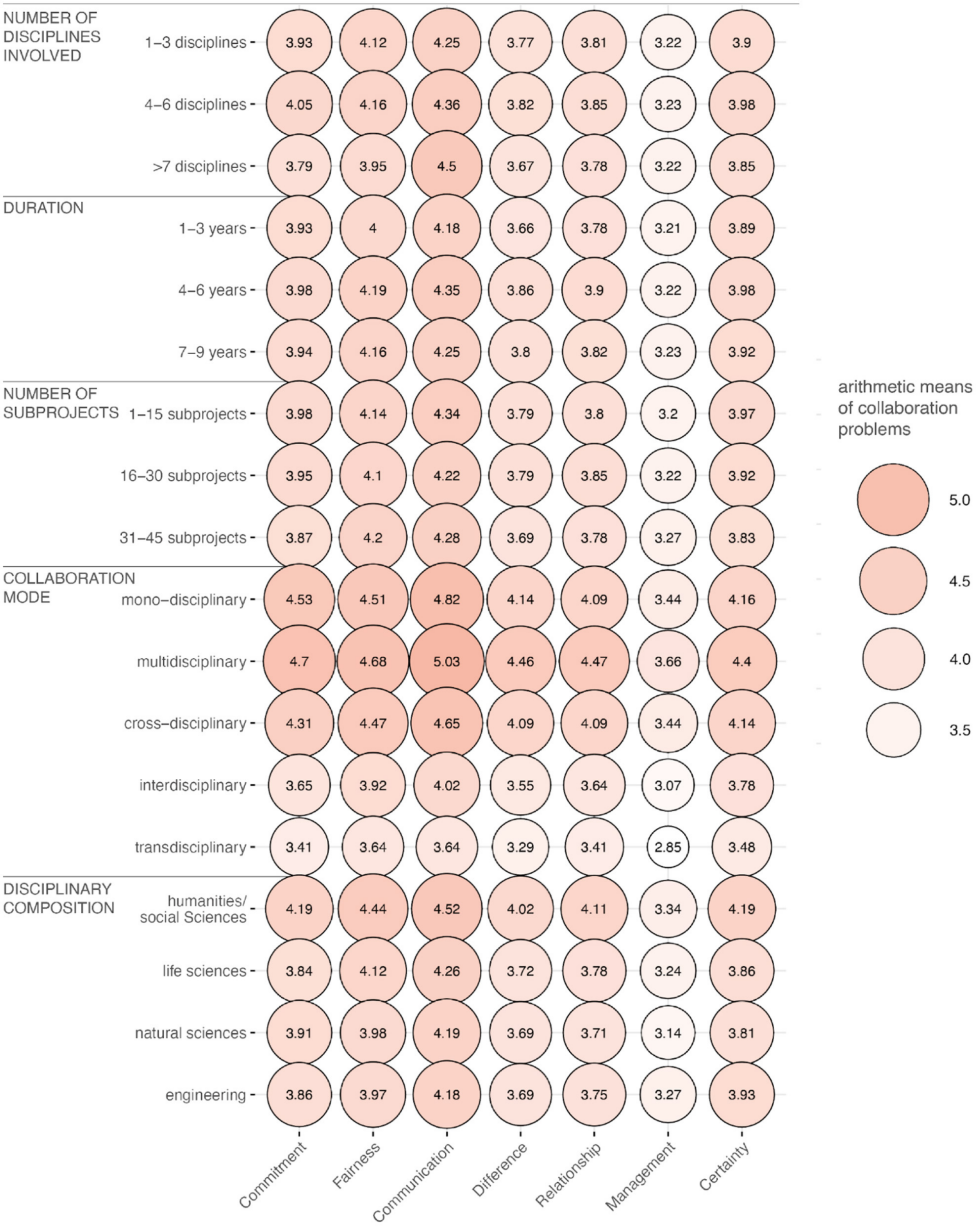
Perceived relevance of the seven collaboration problems differentiated by RC types. The higher the arithmetic mean values of the cumulative indices, the redder and larger the respective balloon. The graph is based on the survey data of *n* = 3,050 PIs and spokespersons after listwise deletion.

Regarding the differences in the perception of collaboration problems depending on different types of RCs (RQ2), the balloon plot (Jain and Warnes, 2006), visualizing the group differences in the mean values, showed only minor deviations from the results for RQ1 (Figure 2). In general, even after the group differentiation, the relevance usually lay at an arithmetic mean value around *M*~ 4. One exception was the management problem, whose relevance was assessed by almost all subgroups with a mean value of *M*~ 3, which was one scale point lower on average than the other problems. The strongest differences in the average relevance rating of the seven problems was apparent when differentiating the RCs according to different modes of collaboration: Those RCs that collaborate in an interdisciplinary or even transdisciplinary manner (see Supplementary Figure A9 in Supplementary material; Kocka, 1987; Jungert et al., 2013) showed, on average, somewhat lower relevance ratings of the seven problems compared to mono-disciplinary, multidisciplinary, or cross-disciplinary RCs.

Considering the visualization of the arithmetic means of the twenty sub-groups through the balloon plot (Figure 2), it can be seen that there are no significant mean differences between the groups for the variables *number of disciplines involved, duration* and *number of sub-projects* (Table 3). Deviating from this, slight group differences emerged regarding the variables *collaboration mode* and *disciplinary composition*: with the exception of the feature combinations *cross-disciplinary vs. mono-disciplinary, cross-disciplinary vs. multidisciplinary*, and *mono-disciplinary vs. multidisciplinary* RCs, all forms of collaboration differed mutually significantly with regard to assessments of the relevance of the problems (Table 4). Additionally, RCs with at least one PI from the humanities/social sciences differed significantly from RCs with at least one PI from the natural, life, or engineering sciences (Table 5). However, as the permutation test for homogeneous variances in the subgroups of the variables *collaboration mode* and *disciplinary composition* was significant in each case (i.e., homogeneous within-group dispersion could not be assumed), both test results should be treated with caution. On the basis of non-homogeneous within-group dispersion, it remains unclear whether the significant differences occurred because there was no homogeneity in the dispersion of within-group distances between the groups or because the groups had significantly different localized centroids in multivariate space (Anderson, 2001).

**Table 3:** PERMANOVA over Euclidean distances based on seven problems for 18 groups of the variables number of disciplines involved, duration, number of subprojects, collaboration mode, and disciplinary composition.

	**df**	**SS**	**MS**	**pseudo F**	***p***
Number of disciplines	2	1.17	0.58	0.56	0.76
Disciplinary composition	3	25.60	8.53	8.50	0.00^**^
Duration	1	0.53	0.52	0.51	0.64
Number of subprojects	3	2.47	0.82	0.80	0.57
Collaboration mode	4	130.39	32.597	33.733	0.00^**^

^**^p < 0.01.

**Table 4:** Pairwise A-posteriori tests between the different collaboration modes.

	**df**	**SS**	**pseudo F**	***p***
Interdisciplinary—cross-disciplinary	1	56.17	63.77	0.01^*^
Interdisciplinary—mono-disciplinary	1	23.75	25.58	0.01^*^
Interdisciplinary—transdisciplinary	1	9.33	10.91	0.01^*^
Interdisciplinary—multidisciplinary	1	53.50	57.57	0.01^*^
Cross-disciplinary—mono-disciplinary	1	0.17	0.16	1.00^*^
Cross-disciplinary—transdisciplinary	1	56.22	61.01	0.01^*^
Cross-disciplinary—multidisciplinary	1	3.63	3.38	0.28^*^
Mono-disciplinary—transdisciplinary	1	36.01	31.95	0.01^*^
Mono-disciplinary—multidisciplinary	1	3.23	2.33	0.91^*^
Transdisciplinary—multidisciplinary	1	61.49	54.82	0.01^*^

^*^p < 0.05. Reported are Bonferroni-corrected *p*-values.

**Table 5:** Pairwise A-posteriori tests between the different disciplinary compositions.

	**df**	**SS**	**pseudo F**	***p***
Humanities/Social Sciences—Natural sciences	1	21.14	20.45	0.01^*^
Humanities/Social Sciences—Life Sciences	1	17.08	16.40	0.01^*^
Humanities/Social Sciences—Engineering	1	5.84	5.31	0.05^*^
Natural sciences—life sciences	1	0.92	0.94	1.00
Natural sciences—engineering	1	0.50	0.52	1.00
Life sciences—engineering	1	0.33	0.34	1.00

^*^p < 0.05. Reported are Bonferroni-corrected p-values.

### 3.3. Differences depending on the characteristics of researchers (RQ3)

**Figure 3:**
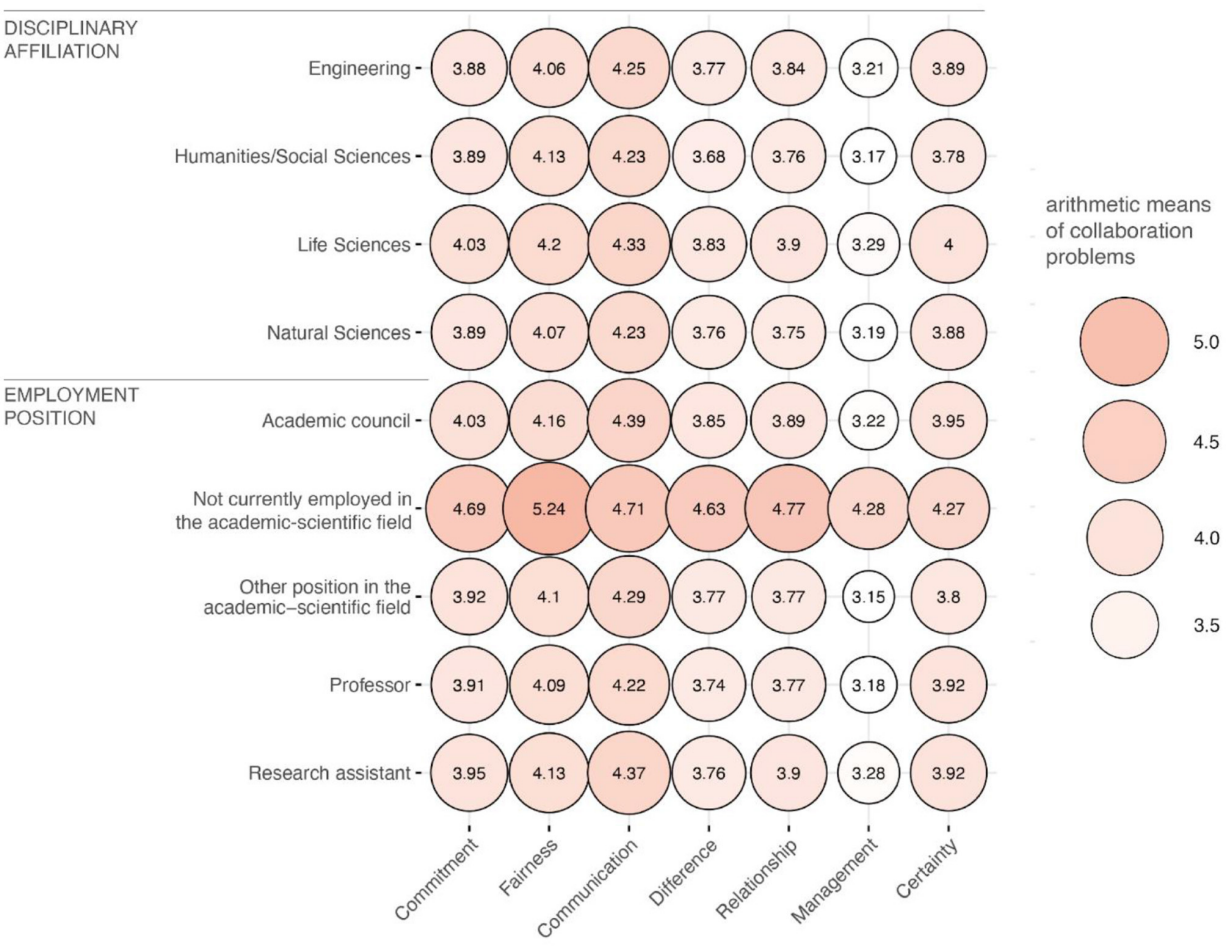
Perceived relevance of the seven collaboration problems differentiated by individual characteristics of researchers. The higher the arithmetic mean values of the cumulative indices, the redder and larger the respective balloon. The graph is based on the survey data of *n* = 3,050 PIs and spokespersons after listwise deletion.

The findings regarding differences in the perception of collaboration problems depending on various individual characteristics of the PIs and spokespersons (RQ3) likewise showed only minor deviations from the results of RQ1 (Figure 3). Even after differentiating the relevance assessments of problems according to *disciplinary affiliation* and *employment position*, the arithmetic mean value mostly lay at around *M*~ 4. An exception is once again the management problem, whose relevance was assessed by almost all subgroups with a mean value of *M*~ 3. The strongest differences in the average relevance assessment of the seven problems can be seen in the differentiation of *employment position*: in particular, PIs and spokespersons who are not employed in the academic-scientific field assessed all seven collaboration problems as having somewhat higher relevance, with an arithmetic mean value of about *M*~ 5.

Accordingly, the PERMANOVA only revealed significant mean value differences between the groups of the variable *current employment position* (Table 6). As the permutation test for homogeneous dispersion for the variable *current employment position* was also not significant—i.e., homogeneous within-group dispersion could be assumed—this finding can be considered as stable. The *post-hoc* test showed that PIs and spokespersons who were not currently employed in the academic-scientific field rated the problems as significantly more relevant than did professors, academic councils, research assistants, and PIs or spokespersons who held another position in the academic-scientific field (Table 7).

**Table 6:** PERMANOVA over Euclidean distances based on seven problems for nine groups of the variables disciplinary affiliation and current employment position.

	**df**	**SS**	**MS**	**pseudo F**	***p***
Disciplinary affiliation	3	5.79	1.92	1.88	0.09
Current employment position	4	9.72	2.4297	2.41	0.01^**^

^**^p < 0.01.

**Table 7:** Pairwise A-posteriori tests between the different employment positions.

	**df**	**SS**	**pseudo F**	***p***
Professor—Academic council	1	0.31	0.30	0.81
Professor—Other position in the academic-scientific field	1	0.47	0.46	0.66
Professor—Research assistant	1	1.04	1.02	0.33
Professor—Not currently employed in the academic-scientific field	1	8.29	8.12	0.00^**^
Academic council—Other position in the academic-scientific field	1	0.32	0.35	0.78
Academic council—Research assistant	1	0.09	0.09	0.99
Academic council—Not currently employed in the academic-scientific field	1	6.23	6.13	0.00^**^
Other position in the academic-scientific field—Research assistant	1	0.46	0.47	0.63
Other position in the academic-scientific field—Not currently employed in the academic-scientific field	1	7.74	7.94	0.00^**^
Research assistant—Not currently employed in the academic-scientific field	1	6.91	6.67	0.00^**^

^**^p < 0.01. Reported are Bonferroni-corrected *p*-values.

### 3.4. Co-occurrence of collaboration problems (RQ4)

In order to model information about the co-occurrence of collaboration problems (RQ4), we specified an MDS based on a distance matrix of Pearson correlations between the cumulative indices of the seven collaboration problems. Although the stress value of the resulting two-dimensional MDS configuration was 0.09, this was significantly smaller than the stress value expected from random data using a significant permutation test (*p* = 0.01). In this respect, the fit of the MDS solution with regard to the specified dimensions and the resulting stress value was therefore acceptable (Borg et al., 2018).

**Figure 4:**
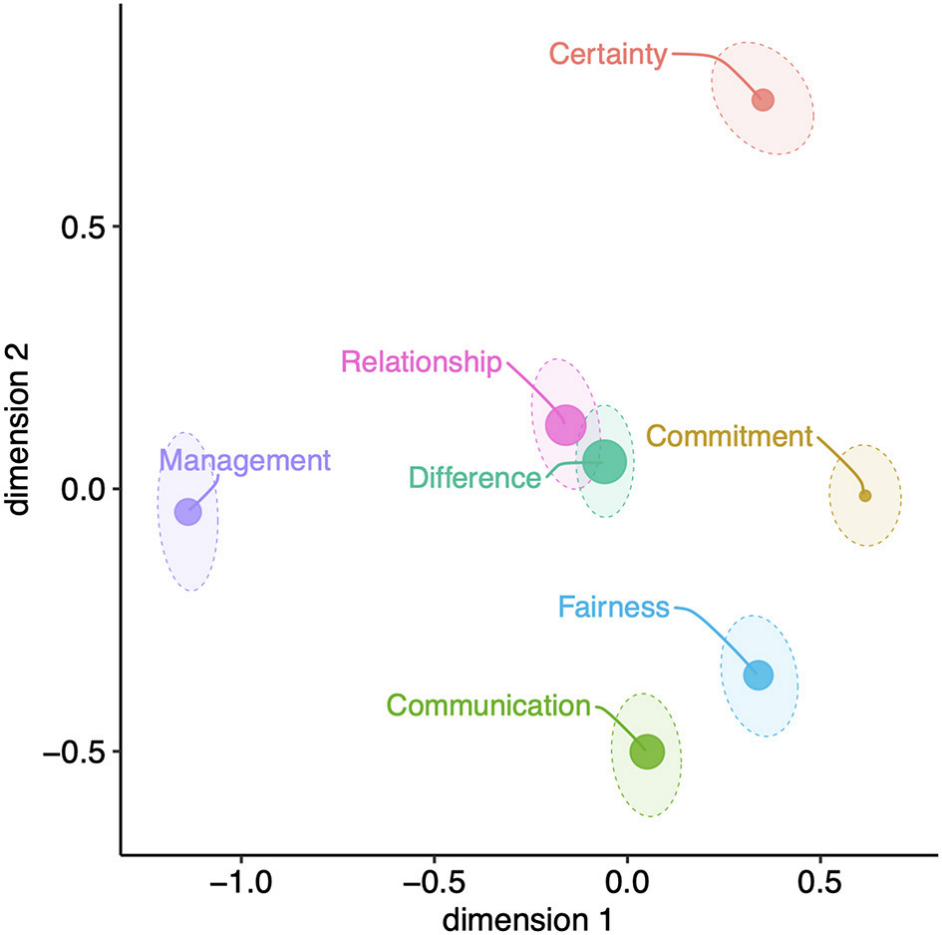
Multidimensional scaling representation of the associations of the seven problems. The dashed ellipses show the 95% confidence intervals of the position of the seven problems. The dot size shows the contribution (stress per point, SPP) of the seven problems to the global stress of the MDS.

To ensure the replicability of the MDS solution and to avoid a premature stop of the iterative adaptation of the MDS at a local minimum of the stress value, different starting configurations of the MDS were specified (Mair et al., 2016). Furthermore, the stability of the MDS solution was tested using bootstrapping (Jacoby and Armstrong, 2014). The dashed ellipses around the indicators in the geometric space of the MDS configuration mark their 95% confidence regions (Figure 4). The more compact a confidence ellipse is, the more likely it is that the true position of the corresponding problem lies on or near the centroid of the respective ellipse. Figure 4 shows that all additive indices have a uniform, rather low uncertainty.

With a total stress value of 0.10, the specified MDS can be classified as acceptable. The stress per point (SPP) value can also be used to quantify the contribution of each input variable to the overall stress value of the MDS. The graph we specified (Figure 4) shows the SPP value of the seven problems based on the size of their localization point. Accordingly, Figure 4 shows that difference problems contribute most to the total stress value and commitment problems contribute least.

The center of the geometric MDS configuration (Figure 4) demonstrates that relationship problems are clearly associated with difference problems. The approximately central position of the relationship and difference problems in the geometric solution of the MDS further indicates that management problems, but especially certainty, commitment, fairness, and communication problems are associated with both relationship and difference problems. In this respect, relationship and difference problems occupy a central position in the network of relationships of the seven collaboration problems: Their occurrence is clearly associated with that of the other five problems. In addition, the lower right corner of the MDS chart shows that communication, fairness, and commitment problems are located in relative proximity to each other, indicating that these three problems are moderately associated with each other. Finally, the problems located at the outer edges of the MDS diagram are again largely uncorrelated with each other: Management and commitment problems as well as certainty and communication problems are thus essentially unrelated.

Overall, the MDS reveals that relationship and difference problems co-occur with management, certainty, commitment, fairness, and communication problems. Furthermore, fairness and communication problems co-occur relatively often. Management, certainty, and commitment problems, on the other hand, are not clearly exclusively associated with another problem dimension.

## 4. Discussion

### 4.1. Summary of the results

The objective of the present study was to gain a deeper understanding of the relative relevance of problems in collaborative research and the interconnections between them. Building on previous insights into the most current problems, we sought to find answers to four major issues, which we addressed through one hypothesis and three research questions: (1) researchers' perceived relative relevance collaboration problems, (2) differences in these perceptions based on the type of RC, (3) and on the characteristics of researchers, and (4) the co-occurrence of collaboration problems.

With respect to issue (1), the results of our representative survey of leading participants of RCs revealed that, broadly speaking, collaboration problems were experienced only to a small degree. Furthermore, we found almost no differences with regard to their perceived relevance in terms of their actual occurrence. The only exception is the management problem, which was experienced to an even smaller degree than the other six problems (i.e., commitment, fairness, communication, difference, relationship, and certainty). This finding is in stark contrast to the qualitative results we relied upon and which, based on a qualitative interview study, hypothesized that difference, commitment, and certainty problems were perceived as more important than the other problems (Meißner et al., 2022). Concerning issues (2) and (3), there were almost no differences concerning the perceived relevance of these problems depending on the type of RC or on the individual researchers. There were three exceptions: First, for all of the seven problems included in the study, non-academics perceived a higher relevance of collaboration problems. Second, collaboration problems occurred less frequently in RCs with a more complex collaboration mode (i.e., trans- and interdisciplinary) and third, in RCs with at least one PI from the humanities/social sciences. Lastly, addressing issue (4), we found close links between relationship and difference problems on the one hand and between fairness and communication problems on the other. The remaining three problems (i.e., commitment, management, and certainty) seem to occur rather independently. Furthermore, relationship and difference problems seem to play a central role in the overall picture of collaboration problems.

### 4.2. Theoretical and practical implications

Taken together, the present findings do not reveal substantial variations regarding the perception of collaboration problems. Some groups generally perceive the seven problems to be more relevant: non-academics, participants of mono-, multi-, and cross-disciplinary RCs, and participants of RCs with members from the humanities/social sciences. While the result that trans- and interdisciplinary RCs perceive a lower relevance of collaboration problems is rather surprising, the other two results can well be explained in our view: First, non-academics might experience more struggles because most RCs might be more adapted to the viewpoints and routines of the academic sphere. Second, because of their scientific background researchers from the humanities/social sciences might be more sensitive to interpersonal problems. However, overall and within each group there is very little variation. Based on our findings, we cannot speak of problems that are of high or medium relevance, but only of less relevant problems. There are several potential reasons for this finding. For example, it might be the case that if research collaboration is perceived as problematic, this could affect all parts and aspects of the collaboration. In turn, this may result in an overall perception of the RC as problematic, in the sense of a halo effect. It might also be possible that researchers have difficulties to trace a problematic collaboration back to a specific problem, especially if confronted with very short items in a questionnaire without the opportunity to carve out a particular problem discursively like in a qualitative interview. Both of these explanations would imply that in practice, researchers cannot easily replicate the clear distinction between specific problems which we proposed in theoretical terms.

However, our results suggest that a problematic collaboration seldom occurs at all. The vast majority of researchers perceived a low relevance of problems, which implies that working in RCs is, at least for the leading personnel, generally experienced as smooth and unproblematic. *Post-hoc* analyses with respect to the overall problem level within the RCs in our sample support this insight: Only 9.7% revealed a high problem level and 1.0% a very high problem level, which in the words of Bozeman and Youtie (2017) can be called “routinely bad” or “nightmare” RCs. Instead, 56.0% may be called “routinely good” and 33.3% even “dream” RCs.^8^ Our study thus draws a more optimistic picture of the nature of collaborative research than one would likely expect. With regard to scientific practice, there seems to be little need for specific measures to address collaboration problems such as training for research leadership. Although this conclusion concurs with previous research (Bozeman and Youtie, 2017), it is rather abstract and should be treated with caution. As the aforementioned figures show, there are individual RCs that struggle with more or less intense problems that may even be likely to put the whole collaboration at risk. It would certainly be worthwhile to take a closer look at “routinely bad” or “nightmare” RCs in order to gain an even more nuanced understanding of collaboration problems and their implications—taking into account the principle that “each unhappy collaboration is unhappy in its own way” (Bozeman and Youtie, 2017). Furthermore, it would be interesting to take a closer look at researchers who are not employed in the academic field, because they are revealed to have a slightly greater problem perception than their colleagues from academia.

Concerning the co-occurrence of specific problems, our findings are also quite plausible. First, the co-occurrence of difference and relationship problems suggests that if differences are perceived as too high, it can be challenging to establish good relationships among the researchers. For example, different working styles may not only lead to conflicts in the workplace but also reduce the motivation to meet socially after work. Put differently, if researchers feel that they need to bridge various forms of differences, this can definitely strain personal relationships, probably even on a daily basis. The co-occurrence of fairness and communication problems is similarly plausible: Insufficient or one-sided communication can certainly result in researchers simply being unaware of their colleagues' work. Consequently, they might get the impression, whether justified or not, of an unfair distribution of individual inputs and outcomes. At the same time, greater communicative effort can be a valuable strategy to overcome any perceptions of unfairness in an RC, i.e., to resolve the fairness problem (Meißner et al., 2022).

### 4.3. Limitations and implications for future research

Our findings can be considered as robust because they rely on a large-scale representative survey, which is rare in this area of research. Nevertheless, some limitations need to be acknowledged. First, we developed the items to measure the collaboration problems based on a qualitative study (Meißner et al., 2022) and did not test these items in advance. Thus, although the item development was theoretically and empirically informed and the items revealed to be valid in analyses apart from this study (Hückstädt, 2022), the construct validity of our measure (Cronbach and Meehl, 1955) needs to be tested in a further study. Second, our sample only included PIs and spokespersons of RCs, and did not include representatives of other roles assumed by researchers in collaborative research, such as doctoral students. It might well be the case that researchers in leadership positions have higher autonomy or power which might lead to different experiences than those of more junior scholars. Specifically, they might overlook or underestimate problems that younger participants in lower hierarchical positions experience. Thus, a more comprehensive sample might report more problems or assess the relevance of some problems differently. Furthermore, with their answers the speakers in our sample actually evaluated themselves, which may have led to certain biases due to a lack of critical self-reflection or honesty. Thus, further research is needed that takes the views of researchers on different career stages into account.

A third limitation concerns the specific RCs that we included in our target population: RCs funded by the German Research Foundation. While the German Research Foundation is the most important and prestigious funding agency in this country and the free accessibility of the data on these RCs provided a good opportunity for our research, our study is limited in geographical terms and does not include RCs funded by other bodies such as the European Union and specific programmes such as H2020. Furthermore, our sample does not include RCs who failed to raise funding or that were not dependent on external funding. Because dsyfunctional RCs might struggle already on the way to get funding and RCs without funding might be less interested in the success of their research, including them in the sample would probably lead to different results. Therefore, future research needs to explore whether our findings can be generalized beyond this specific sample. Lastly, our findings rely on self-reported data, bringing the usual issues: The respondents may not have been aware of collaboration problems or may have been unwilling to admit that their RC is not actually a “dream” one. Therefore, our findings need to be validated through more objective parameters such as bibliometric measures in order to produce findings that may be used to guide policy decisions. For example, a RC might experience itself as unproblematic but its productivity in terms of published papers might suggest otherwise.

## 5. Conclusions

The present study took a closer look at problems in collaborative research. From our perspective, the empirical results are rather surprising. In theoretical and practical terms, we can conclude that overall, collaboration problems are less prevalent and relevant than one might assume. Furthermore, because these insights could only be gained through a close combination of expert interviews (Meißner et al., 2022) and survey data, our study provides an example of a fruitful triangulation of qualitative and quantitative methods. Generally speaking, our study underlines that “most research teams are ‘happy ones”' (Bozeman and Youtie, 2017). The challenge is now to underpin and differentiate these insights through further studies, using different samples (e.g., postdocs) and methodological approaches (e.g., bibliometric analyses).

## Data availability statement

The datasets presented in this article are not readily available because of privacy restrictions. Requests to access the datasets should be directed to CW, c.weinmann@hhu.de.

## Ethics statement

Ethical review and approval was not required for the study on human participants in accordance with the local legislation and institutional requirements. The patients/participants provided their written informed consent to participate in this study.

## Author contributions

MH, FM, and GV contributed to conception and design of the study. MH organized the database and performed the statistical analysis. CW wrote the first draft of the manuscript. MH and FM wrote sections of the manuscript. All authors contributed to manuscript revision, read, and approved the submitted version.

## Footnotes

^1^Specifically, these are *Research Units, Research Centers, Priority Programmes, Collaborative Research Centers* and *Transregios*.

^2^The spectrum of possible (inter-)disciplinary cooperation can be represented as a continuum, with disciplinary cooperation as the negative pole and transdisciplinary cooperation as the positive pole. Disciplinary cooperation is characterized by the fact that it remains within its own boundaries in the context of knowledge production: There is no cross-disciplinary exchange and no cross-disciplinary integration of research results. In the context of transdisciplinary cooperation, on the other hand, there is a continuous and high level of cross-disciplinary exchange as well as a cross-disciplinary integration of disciplinary research results, with the additional goal of generating application-oriented knowledge. Multi-, cross-, as well as interdisciplinary research collaborations move between these two poles (Keestra and Menken, 2017; Supplementary Figure A9 in Supplementary material).

^3^For the distributions of the corresponding variables, see Supplementary Tables A1–A7 in the Supplementary material.

^4^The disciplines involved in RCs were queried according to the subject structure of the German Research Foundation (2023a).

^5^More than 99% of all respondents participated in their projects for more than one year, which is why we did not take the duration of participation as a variable into account.

^6^The following comments refer to the case in which all objects in the input material of the MDS model are positively correlated (Borg et al., 2018).

^7^We assume that the value 4 in an additive index with a possible range of 2–10 can be interpreted as rather low. From the authors' point of view, however, there is no objective cut-off value for problems to be considered low, medium, or high.

^8^For these analyses, we classified the RCs into four groups, using the terminology of Bozeman and Youtie : (1) “dream” (overall problem level: 7–22), (2) “routinely good” (overall problem level: 23–38), (3) “routinely bad” (overall problem level: 39–54), and (4) “nightmare” (overall problem level: 55–70). The percentages do not add up to 100% because not all participants answered these questions.
