# Supplementary material for: How do researchers perceive problems in research collaboration? Results from a large-scale study of German scientists
Source: Front Res Metr Anal. 2023 Feb 23;8:1106482. doi: 10.3389/frma.2023.1106482 (PMC9997842; doi:10.3389/frma.2023.1106482)

Figure A6

Duration of the Research Cluster in Years According to Funding Line: Research Units, Research Centres, Clusters of Excellence, Collaborative Research Centres, Transregios and Priority Programmes

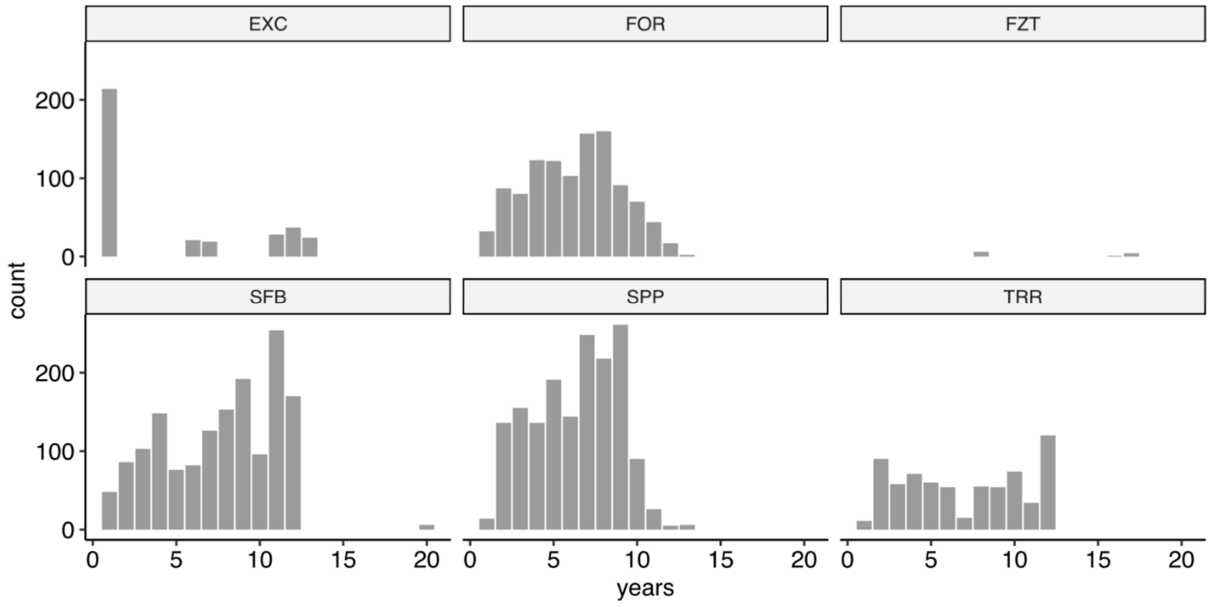

Supplement: Supplementary file 6 [file Image_6.pdf]
