# Supplementary material for: How do researchers perceive problems in research collaboration? Results from a large-scale study of German scientists
Source: Front Res Metr Anal. 2023 Feb 23;8:1106482. doi: 10.3389/frma.2023.1106482 (PMC9997842; doi:10.3389/frma.2023.1106482)

Figure A7

Relative Frequencies of the Disciplinary Affiliation of the PIs and Spokespersons in the Population and the Sample

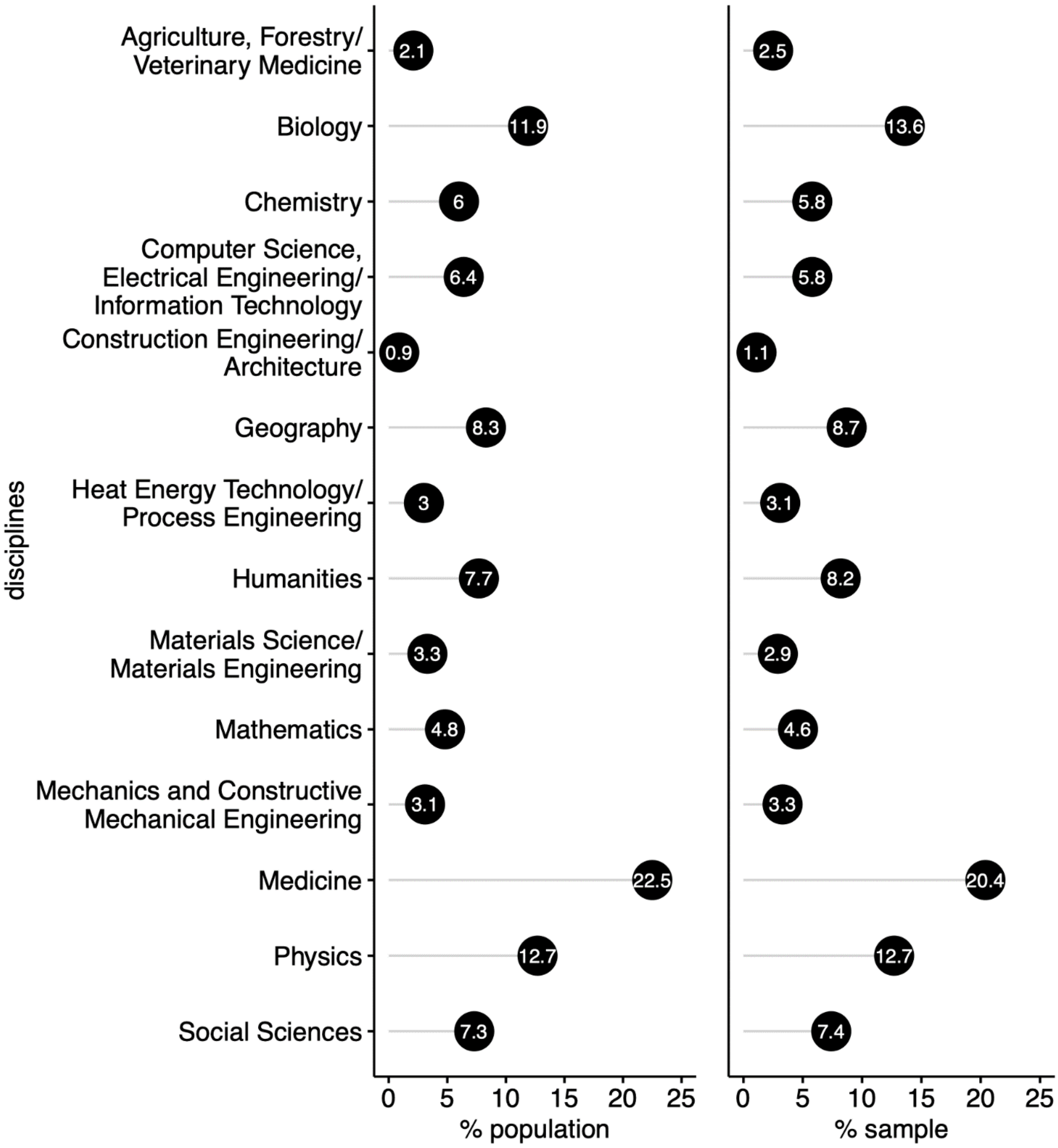

Supplement: Supplementary file 7 [file Image_7.pdf]
