# Supplementary material for: How do researchers perceive problems in research collaboration? Results from a large-scale study of German scientists
Source: Front Res Metr Anal. 2023 Feb 23;8:1106482. doi: 10.3389/frma.2023.1106482 (PMC9997842; doi:10.3389/frma.2023.1106482)

Figure A8

Relative Frequency of Researchers' Gender and Status Roles, RC status and Their Funding Line in the Population and in the Sample

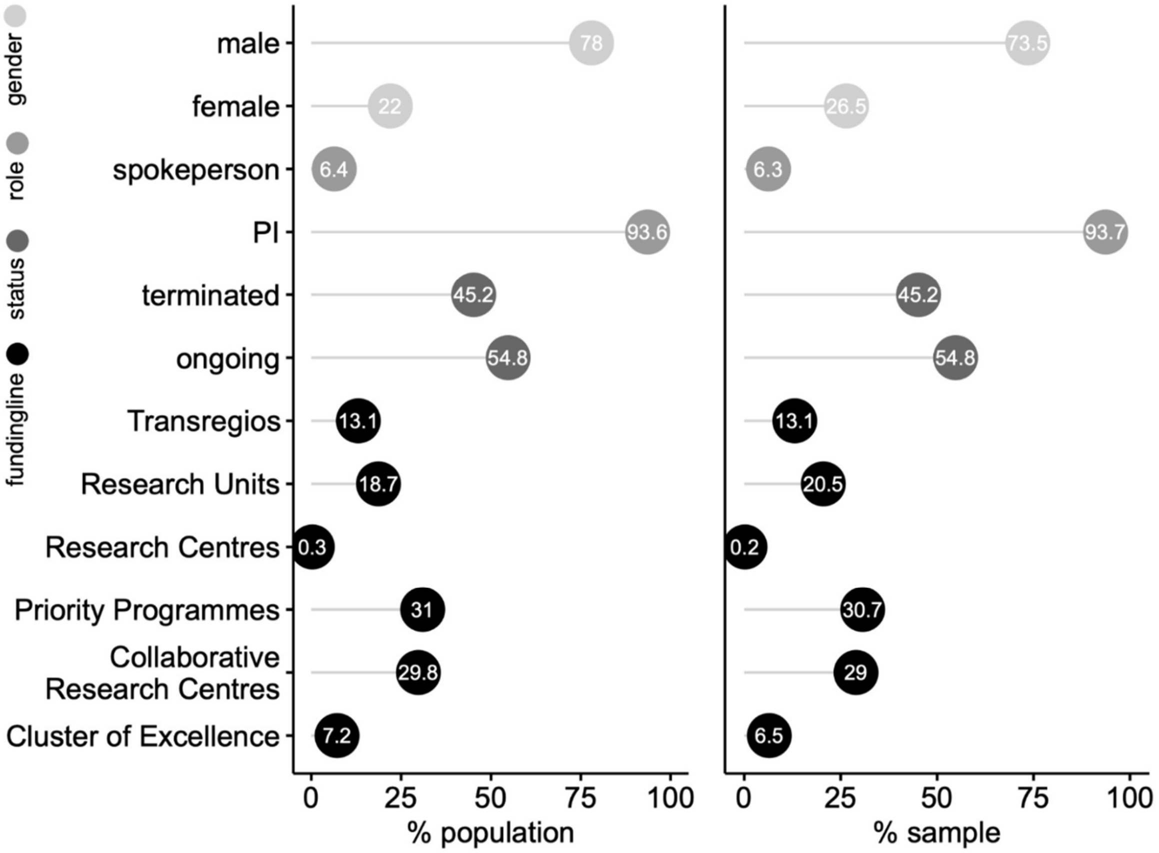

Supplement: Supplementary file 8 [file Image_8.pdf]
