# Supplementary figures and images for: How do researchers perceive problems in research collaboration? Results from a large-scale study of German scientists
Source: Front Res Metr Anal. 2023 Feb 23;8:1106482. doi: 10.3389/frma.2023.1106482 (PMC9997842; doi:10.3389/frma.2023.1106482)

**Figure A9***Forms of Collaboration (Keestra and Menken, 2017)*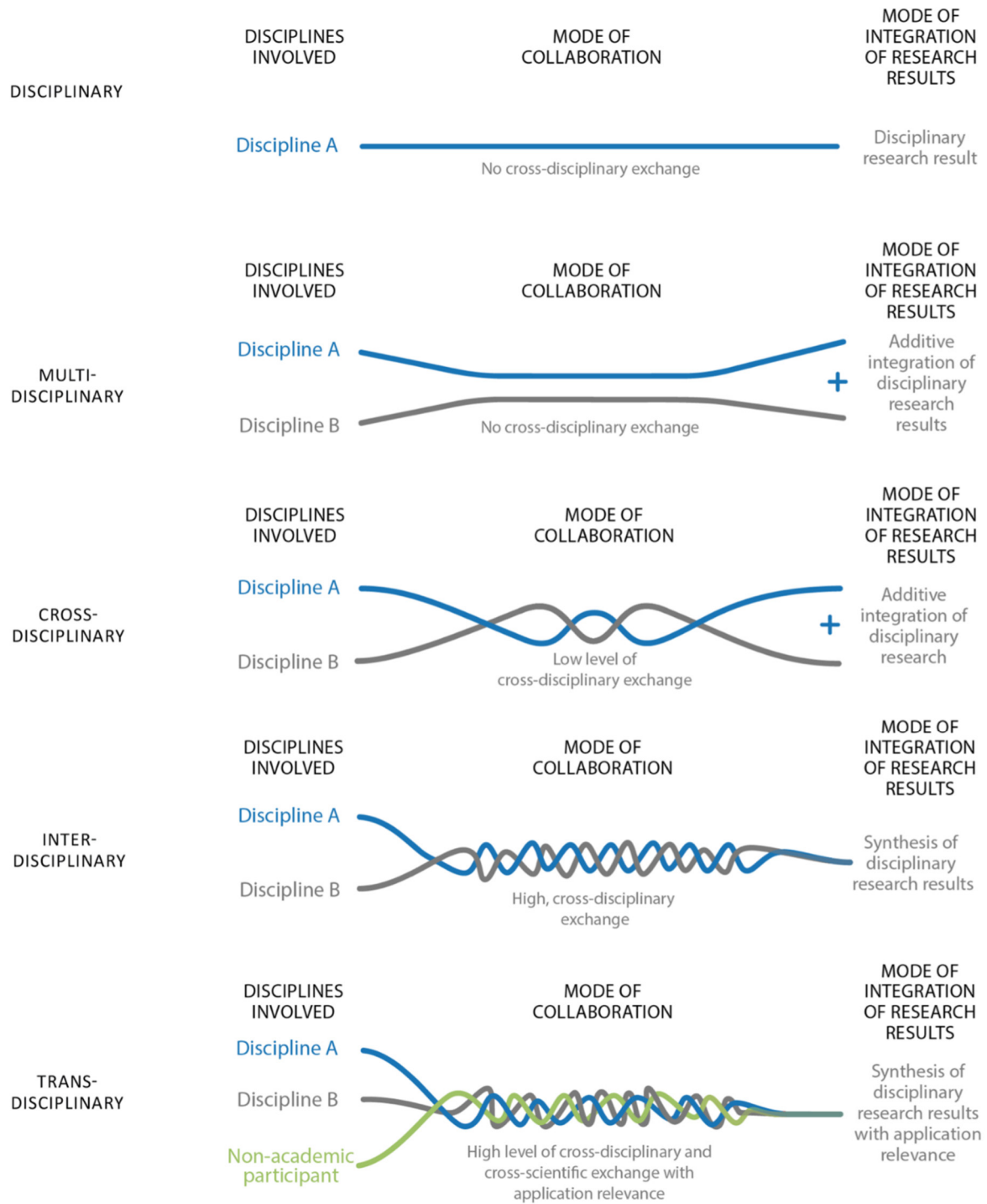

Supplement: Supplementary file 9 [file Image_9.pdf]
