# Supplementary material for: How do researchers perceive problems in research collaboration? Results from a large-scale study of German scientists
Source: Front Res Metr Anal. 2023 Feb 23;8:1106482. doi: 10.3389/frma.2023.1106482 (PMC9997842; doi:10.3389/frma.2023.1106482)
Supplement: Supplementary file 10 [file Table_1.docx]

| **Table A1** *Number of Disciplines Involved* | | | |
| --- | --- | --- | --- |
| 1–3 disciplines | 4–6 disciplines | ≥ 7 disciplines | missings |
| 4387 | 758 | 61 | 120 |
