# Supplementary material for: How do researchers perceive problems in research collaboration? Results from a large-scale study of German scientists
Source: Front Res Metr Anal. 2023 Feb 23;8:1106482. doi: 10.3389/frma.2023.1106482 (PMC9997842; doi:10.3389/frma.2023.1106482)
Supplement: Supplementary file 11 [file Table_2.docx]

| **Table A2** *Duration* | | | |
| --- | --- | --- | --- |
| 1–3 years | 4–6 years | ≥ 7 years | missings |
| 1114 | 1331 | 2298 | 583 |
