# Supplementary material for: How do researchers perceive problems in research collaboration? Results from a large-scale study of German scientists
Source: Front Res Metr Anal. 2023 Feb 23;8:1106482. doi: 10.3389/frma.2023.1106482 (PMC9997842; doi:10.3389/frma.2023.1106482)
Supplement: Supplementary file 12 [file Table_3.docx]

| **Table A3** *Number of Sub-projects* | | | | | |
| --- | --- | --- | --- | --- | --- |
| 1–10 subprojects | 11–20 subprojects | 21–30 subprojects | 31–40 subprojects | > 40 subprojects | Missings |
| 694 | 1419 | 2013 | 595 | 244 | 361 |
