# Supplementary material for: How do researchers perceive problems in research collaboration? Results from a large-scale study of German scientists
Source: Front Res Metr Anal. 2023 Feb 23;8:1106482. doi: 10.3389/frma.2023.1106482 (PMC9997842; doi:10.3389/frma.2023.1106482)
Supplement: Supplementary file 13 [file Table_4.docx]

| **Table A4** *Collaboration Mode* | | | | | |
| --- | --- | --- | --- | --- | --- |
| Disciplinary | Multi-disciplinary | Cross-disciplinary | Inter-disciplinary | Trans-disciplinary | Missings |
| 403 | 403 | 958 | 2064 | 319 | 1179 |
