# Supplementary material for: How do researchers perceive problems in research collaboration? Results from a large-scale study of German scientists
Source: Front Res Metr Anal. 2023 Feb 23;8:1106482. doi: 10.3389/frma.2023.1106482 (PMC9997842; doi:10.3389/frma.2023.1106482)
Supplement: Supplementary file 14 [file Table_5.docx]

| **Table A5** *Disciplinary Composition of the RC (Involvement of at Least One PI oder Spokeperson from the Above-mentioned Subjects)* | | | | |
| --- | --- | --- | --- | --- |
| Humanities/social sciences | Life sciences | Natural sciences | Engineering | Missings |
| 1068 | 1569 | 1674 | 231 | 784 |
