# Supplementary material for: How do researchers perceive problems in research collaboration? Results from a large-scale study of German scientists
Source: Front Res Metr Anal. 2023 Feb 23;8:1106482. doi: 10.3389/frma.2023.1106482 (PMC9997842; doi:10.3389/frma.2023.1106482)
Supplement: Supplementary file 15 [file Table_6.docx]

| **Table A6** *Discilinary affiliation of the PIs and spokepersons* | | | | |
| --- | --- | --- | --- | --- |
| Humanities/social sciences | Life sciences | Natural sciences | Engineering | Missings |
| 828 | 1934 | 1690 | 856 | 18 |
