# Supplementary material for: How do researchers perceive problems in research collaboration? Results from a large-scale study of German scientists
Source: Front Res Metr Anal. 2023 Feb 23;8:1106482. doi: 10.3389/frma.2023.1106482 (PMC9997842; doi:10.3389/frma.2023.1106482)
Supplement: Supplementary file 16 [file Table_7.docx]

| **Table A7** *Employment Position* | | | | | |
| --- | --- | --- | --- | --- | --- |
| Academic council | Currently no employment in the academic-scientific field | Other position in the academic-scientific field | Professor | Research assistant | Miissings |
| 207 | 32 | 368 | 2344 | 614 | 1761 |
